# Electromagnetic Properties of Natural Plant Leaves for Eco-Friendly and Biodegradable Substrates for Wireless IoT Devices

**DOI:** 10.3390/s25041118

**Published:** 2025-02-12

**Authors:** Nikolay Todorov Atanasov, Blagovest Nikolaev Atanasov, Gabriela Lachezarova Atanasova

**Affiliations:** 1 Department of Communication and Computer Engineering, South-West University “Neofit Rilski”, 2700 Blagoevgrad, Bulgaria; gatanasova@swu.bg; 2 Faculty of Telecommunications, Technical University of Sofia, 1000 Sofia, Bulgaria; b_atanasov@outlook.com

**Keywords:** substrate biodegradable, precision agriculture, IoT, substrate for flexible eco-friendly plant wearable antennas, plant permittivity, corn permittivity, wheat permittivity, sunflower permittivity, substrate for plant health sensors

## Abstract

Today, innovative engineering solutions, including IoT devices, enable the precise monitoring of plant health and the early detection of diseases. However, the lifespan of IoT devices used for the real-time monitoring of environmental or plant parameters in precision agriculture is typically only a few months, from planting to harvest. This short lifespan creates challenges in managing the e-waste generated by smart agriculture. One potential solution to reduce the volume and environmental impact of e-waste is to use more environmentally friendly and biodegradable materials to replace the non-degradable components (substrates) currently used in the structure of IoT devices. In this study, we estimate the electromagnetic properties at 2565 MHz of the leaves from three widely grown crops: winter wheat, corn, and sunflower. We found that winter wheat and sunflower leaves have values of the real part of relative permittivity ranging from about 33 to 69 (wheat) and 13 to 32 (sunflower), respectively, while corn exhibits a value of about 33.5. Our research indicates that the position of a leaf on the plant stem and its distance from the soil significantly affect the relative permittivity of winter wheat and sunflower. These relationships, however, are not evident in the electromagnetic properties of corn leaves.

## 1. Introduction

Today, the Internet of Things (IoT), assisted by wireless technologies (cellular and non-cellular), provides innovative engineering solutions across multiple sectors (from healthcare to agriculture) for the effective improvement of the quality of services, and even the quality of agricultural products [1,2,3]. GSMA [2] and IoT Analytics [4] forecast that the global IoT market will expand, and the number of connected IoT devices are anticipated to grow approximately twice, from 18.8 billion at the end of 2024 to about 40 billion by 2030. Although digital transformation offers numerous benefits to various sectors, the rise in electronic devices, particularly IoT devices, contributes to increased electronic waste (e-waste) becoming a threat to the environment [5,6,7]. Furthermore, the lifespan of IoT devices used for real-time monitoring of environmental or plant parameters in precision agriculture is typically only a few months (from planting in the agricultural field to harvesting the crop). This short lifespan presents new challenges in managing e-waste generated by ‘smart’ agriculture. A significant issue is the collection and disposal of used sensors, which are non-degradable. One solution to reduce the volume and environmental impact of e-waste is to use more environmentally friendly materials to replace the non-degradable components currently used in the structure of IoT devices [8,9,10].

**Figure 1:**
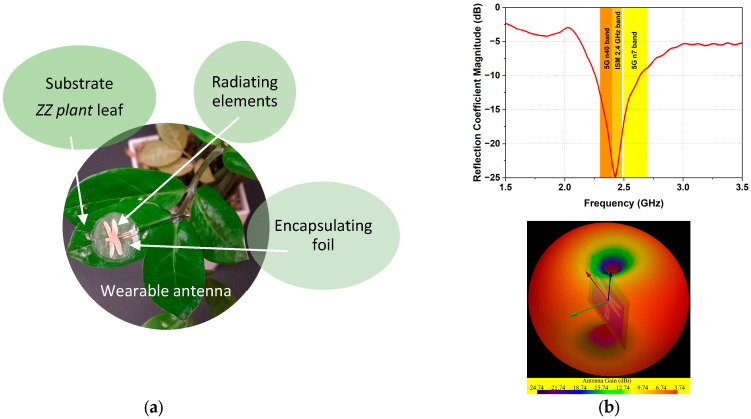
Application example of a wearable antenna on a substrate from a *ZZ plant* leaf: (**a**) photo of the antenna prototype; (**b**) measured reflection coefficient |S_11_| and 3D radiation pattern.

Precision agriculture utilizes a variety of IoT devices. Among the most promising for monitoring plant health and early disease detection are wearable IoT devices that can be attached to a plant’s leaf, stem, or even root [1,11,12]. These plant wearable devices have several components, including a substrate, sensor, microprocessor, transmitter, and antenna. The substrate is essential for the device’s structure, providing a stable platform for other components such as sensors and antenna. Organic materials, such as plant leaves, have been identified as suitable substrates for wearable plant devices [1,6,11,13,14]. Figure 1a shows an example of using a leaf of the *ZZ plant as* an eco-friendly substrate for a wearable antenna for plant health monitoring. As illustrated in Figure 1a, the antenna consists of a dragonfly-shaped radiating element fed by a coplanar waveguide, fully encapsulated by a transparent polymer foil, placed on a biodegradable substrate (a leaf of the ZZ plant). The antenna achieves a wide frequency bandwidth covering ISM 2.45 GHz, 5G bands 7 and 8, and an omnidirectional radiation pattern (Figure 1b). These characteristics make it an excellent candidate for IoT-based plant health monitoring applications in smart agriculture. Additional information regarding the structure and performance of the antenna can be found in reference [1].

Moreover, accurately designing and evaluating the performance of a plant wearable device, particularly a plant wearable antenna, requires knowing the electromagnetic properties of the materials (plant leaves) at the relevant frequency [9]. Furthermore, understanding the dielectric properties of plants is crucial for developing new sensors and models of electromagnetic wave propagation in agricultural fields under various scenarios of IoT-based smart agriculture [15]. Research has shown that the electromagnetic properties of certain agricultural products, such as shelled corn, grain, and soybeans, vary depending on factors like moisture content, frequency, and temperature [16,17]. Also, the electromagnetic properties of fresh leaves from different plant species have been studied and presented [9,18,19,20]. The results show that the complex dielectric permittivity varies depending on the plant species. There is limited knowledge about changes in electromagnetic properties, such as complex permittivity and loss tangent, during plant growth at microwave frequencies. Information about the electromagnetic properties of corn, wheat, and sunflower leaves from real agriculture fields is also limited.

This study aims to investigate the electromagnetic properties at 2565 MHz (ISM band) of the leaves of three widely grown crops: (i) winter wheat, (ii) corn, and (iii) sunflower for applications as substrates for plant wearable sensors and antennas. Additionally, it aims to examine the changes in electromagnetic properties during winter wheat growth, applying this knowledge to the design stage in developing an IoT plant wearable device.

## 2. Materials and Methods

### 2.1. Materials

#### 2.1.1. Plant Material and Sampling Sites

**Figure 2:**
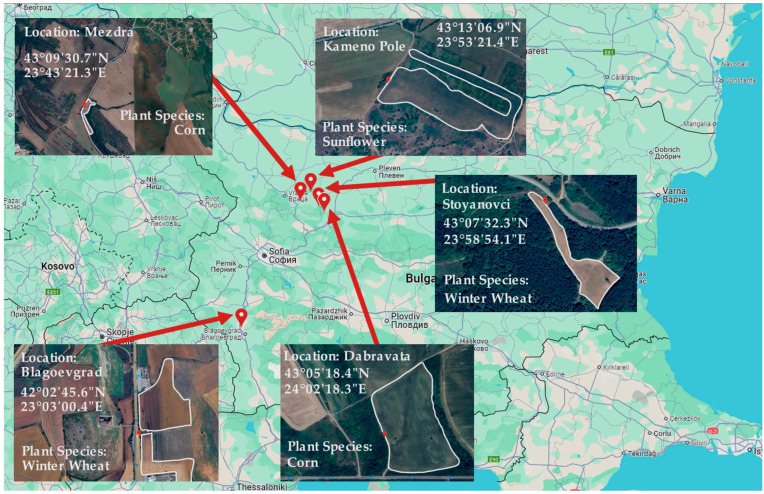
Locations of agricultural fields and their GPS coordinates.

For the study, we collected leaves from three different plant species (corn, winter wheat, and sunflower) from five agricultural fields in Bulgaria. Figure 2 illustrates the GPS coordinates of the field and the plant species.

To investigate the variation in electromagnetic parameters in individual leaves, we collected samples from all green leaves on the stems of 10 corn plants at the R5 growth stage. Moreover, to study the impact of the environment, we took samples from two agricultural fields near Mezdra and Dabrava in Bulgaria (Figure 2). We also investigate the variation in electromagnetic parameters of all green leaves from a sunflower plant to analyze the variation in electromagnetic parameters in plant leaves.

Additionally, we collected samples of winter wheat leaves from 3 April to 8 May 2024, to investigate how electromagnetic parameters in plant leaves vary during growth. All samples were taken from an agricultural field near Blagoevgrad (see Figure 2) every three weeks. During the study, the wheat was in growth stages according to the Feekes scale, from Feekes 8 to Feekes 10.5. Additionally, to assess the impact of the environment on the electromagnetic parameters of wheat, we collected samples of winter wheat from another agricultural field (near Stoyanovtci, Figure 2), where the plants were at the Feekes 7 stage.

#### 2.1.2. Sample Preparation

**Figure 3:**
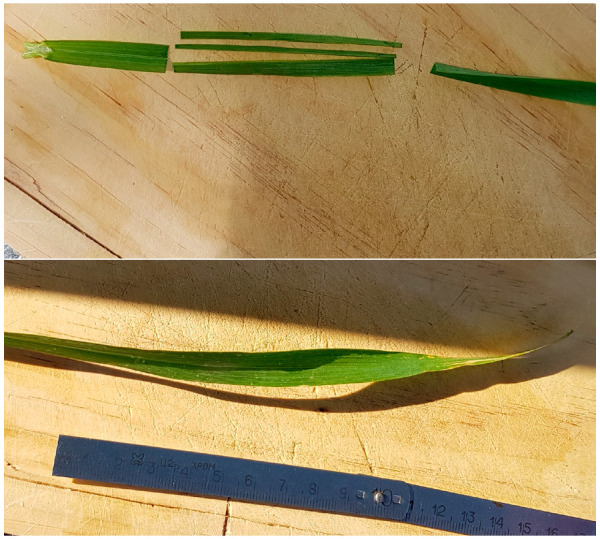
Photos of the sample preparation procedure.

To prepare the sample, we detached a leaf from the winter wheat in the agriculture field and placed it on a wooden plate. Next, we used a blade to cut a rectangular sample from each leaf, as shown in Figure 3. Before cutting the samples, we measured and recorded leaf length, width, and thickness. Each sample is prepared immediately before measuring its electromagnetic parameters. We used the same procedure to prepare the samples from corn and sunflowers. Moreover, for each measurement, soil relative humidity was determined using a Testo 440 dP (Testo Ltd., Alton, UK) and a robust humidity/temperature probe (high-precision temperature/humidity probe 0636 9775, Testo Ltd., UK).

### 2.2. Methods and Experimental Setup

#### 2.2.1. Cavity Perturbation Method

In this study, we measured the electromagnetic properties of the plant leaves at a frequency of 2565 MHz using the cavity perturbation method. We chose this frequency because it is very close to the range of 2400 MHz to 2480 MHz, dedicated to industrial, scientific, and medical (ISM) applications according to existing ITU, ECC, and FCC regulations. ISM is one of the frequency ranges most used by wireless technologies for smart agriculture, including standards such as IEEE 802.11 [21], IEEE 802.15.1 (Bluetooth) [22], IEEE 802.15.4 (Zigbee) [23], etc.

We chose the cavity perturbation method due to its low uncertainties [24]. For the measurement of real (*ε_r_*′) and imaginary (*ε_r_*″) parts of complex permittivity, we introduced the sample from a fresh leaf into a rectangular cavity at the position of the maximum intensity of the electric field. As it is known from theory, placing a small sample into the resonant cavity causes a frequency shift and a decrease in the quality factor compared to an empty resonator [24]. From here, through Equations (1) and (2), we analytically calculated the complex permittivity of the fresh leaf sample:

*ε_r_*′ = (((*f_cr_* − *f_ls_*)/2*f_ls_*) × (*V_cr_* − *V_ls_*)) + 1, (1)

where *V_cr_* and *V_ls_* are the volumes of the cavity resonator and sample, and *f_cr_* and *f_ls_* are resonant frequency measured before (empty cavity) and after the insertion of fresh leaf sample into the resonator.

ε_*r*_″ = (*V_cr_*/4*V_ls_*) × ((1/*Q_ls_*) − (1/*Q_cr_*)), (2)

where *V_cr_* and *V_ls_* are the volumes of the cavity resonator and sample, and *Q_cr_* and *Q_ls_* are quality factors measured before and after the sample insertion into the resonator.

#### 2.2.2. Experimental Setup

**Figure 4:**
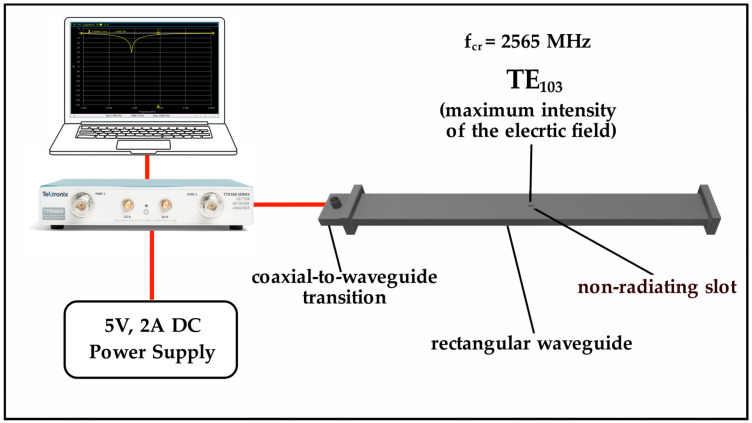
Block diagram of the experimental setup.

Figure 4 depicts the block diagram of the experimental setup. To measure the complex permittivity of fresh leaves, we used a TE_103_ rectangular cavity resonator (with dimensions in millimetres 610.0 × 61.0 × 10.0) formed by a section of rectangular waveguide with short-circuited one end and an iris by the other end to connect to the coaxial–waveguide transition. To introduce the fresh leaf sample, we created two small non-radiating slots on the top and bottom surfaces of the cavity walls, positioning them at the location of the maximum intensity of the electric field. We connect a vector network analyzer (Tektronix TTR503A, Tektronix, Inc., Beaverton, OR, USA) to the coaxial waveguide transition to measure the S_11_ parameter and determine the resonant frequency. Before measuring S_11_, we calibrate the experimental setup by applying the Open-Short-Load calibration technique. This involved utilizing a Short, an Open, and a 50-ohm Load standard to eliminate any residual impedance from the cable. We establish the resonant cavity at the critical coupling. Additionally, we validate the experimental setup by measuring the complex permittivity of Teflon, obtaining measured values for the real and imaginary parts of the relative complex permittivity as 1.97 ± 0.02 and 0.0051 ± 0.0003, respectively. For the measurement of *ε_r_*′ and *ε_r_*″ of samples, we used the measurement procedure described in Section 2.2.1.

Before conducting measurements to determine the electromagnetic properties of plants, we identified the potential sources of systematic and random errors. We divided these error sources into two groups: those related to the sampling process and those associated with the cavity perturbation method and used equipment. We found that sampling plant leaves can introduce random errors and significantly impact the results. Hence, we standardized the leaf samples by cutting them to the same size to minimize this random error. To reduce the random errors arising from human observation of frequencies during the measurement process, we wrote a Python code (version 3.12.1) to determine the frequency of interest from the vector network analyzer. To reduce the systematic errors arising from connecting the components of a measurement system, we calibrated the experimental setup before measuring S_11_. Moreover, the change in environmental conditions surrounding the measurement system also can be a source of random errors. Taking repeated measurements helps to reduce this type of error.

## 3. Results and Discussion

### 3.1. Electromagnetic Parameters of Winter Wheat Leaves

The research in this subsection focuses on the evaluation of the electromagnetic properties of winter wheat leaves during growth.

**Figure 5:**
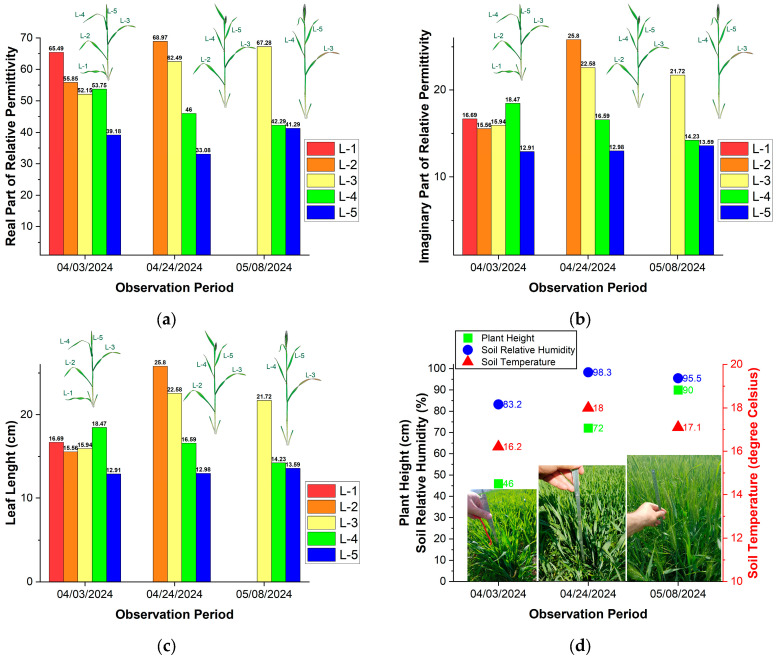
Results from measurements of winter wheat leaves during three growth stages (from Feekes 8 to Feekes 10.5) in the agriculture field near Blagoevgrad: (**a**) Real part of the relative permittivity; (**b**) Imaginary part of the relative permittivity; (**c**) Leaf length; (**d**) Plant height, soil relative humidity and temperature.

Figure 5a,b depict the results of measured real and imaginary parts of the relative permittivity in winter wheat leaves at three stages during growth (from Feekes 8 to Feekes 10.5) in the agriculture field near Blagoevgrad (see Figure 2). Figure 5c,d illustrate the height of plants and the length of winter wheat leaves during their growth. In Figure 5a–c, the leaves are labelled according to their position on the plant stem and relative to the soil (i.e., the first leaf close to the soil is L-1, the second leaf above it is L-2, and so forth). We used the same leaf number (L-1, L-2, etc.) throughout the observation period. For partially dried or dried leaves, permittivity is not measured.

During the observation period of the wheat growth and development, its structure changes continuously. The first (L-1) and second (L-2) leaves die (dried), the stem elongates, and the flag leaf emerges and develops. At the end of the observation period, the head is fully developed (see Figure 5d).

Figure 5 shows that the position of the leaf relative to the soil significantly influences the real part of the relative permittivity. During the measurements on April 3, we observed that L-1 has a value of *ε_r_*′ = 65.49. The results indicated that on April 24, was recorded the highest value for *ε_r_*′ = 68.97 for L-2. At this time, L-1 had withered, while L-2 was the green leaf closest to the soil. Additionally, the analysis of results from May 8 showed that L-3 has the highest value at *ε_r_*′ = 67.28. By this stage of development, both L-1 and L-2 had withered, and L-3 was the green leaf nearest to the soil. Therefore, we can conclude that the green leaf closest to the soil tends to have the highest value of the real part of the relative permittivity. The results indicated that the topmost leaf (L-5) had the lowest values of *ε_r_*′ from 39.18, 33.08, and 41.29 throughout the observation period.

The results may be related to the vertical leaf nitrogen and chlorophyll content distribution within winter wheat. A significant difference in nitrogen and chlorophyll content vertical distribution patterns for winter wheat was found in [25,26], demonstrating that the nitrogen and chlorophyll content in the leaves decreased from top to bottom. The bottom leaves are responsible for the transferability of nitrogen to the top leaf [27]. Thus, the variations in the real part of the relative permittivity of wheat leaves are likely due to the different distribution of nitrogen, chlorophyll, and nutrient content in the upper and lower layers of the leaves.

Figure 5b indicates that the imaginary part of the relative permittivity fluctuates between 12.91 and 25.8 throughout the observation period. On April 24 (L-2) and May 8 (L-3), the green leaf closest to the soil tends to have the highest value of the imaginary part of the relative permittivity. At a soil relative humidity of 83.23%, *ε_r_*″ varies between 12.91 and 18.47, while at a soil relative humidity of 98.3%, it varies from 12.98 to 25.80. The results may be related to vertical water content distribution within winter wheat leaves found by Kong et al. [26], namely that leaf water content within winter wheat canopies tended to be higher in the middle layer and lower in the upper and bottom leaf layers.

**Figure 6:**
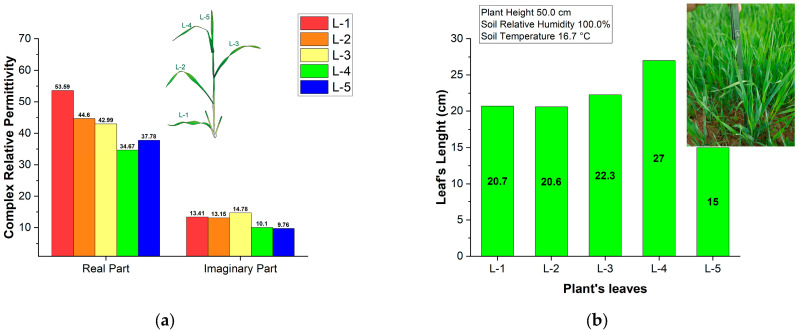
Results from measurements of winter wheat leaves in the agriculture field near Stoyanovtci: (**a**) Real and imaginary parts of the relative permittivity; (**b**) Leaf length, plant height, soil relative humidity and temperature.

To test our hypothesis, we measured the electromagnetic parameters of winter wheat leaves in an agricultural field near Stoyanovtci (see Figure 2). The values of the real and imaginary parts of relative permittivity represented in Figure 6 are similar to those of Figure 5, which proves our hypothesis.

### 3.2. Electromagnetic Parameters of Corn

The research in this subsection focuses on the evaluation of the electromagnetic properties of corn leaves in harsh environmental conditions, specifically drought.

**Figure 7:**
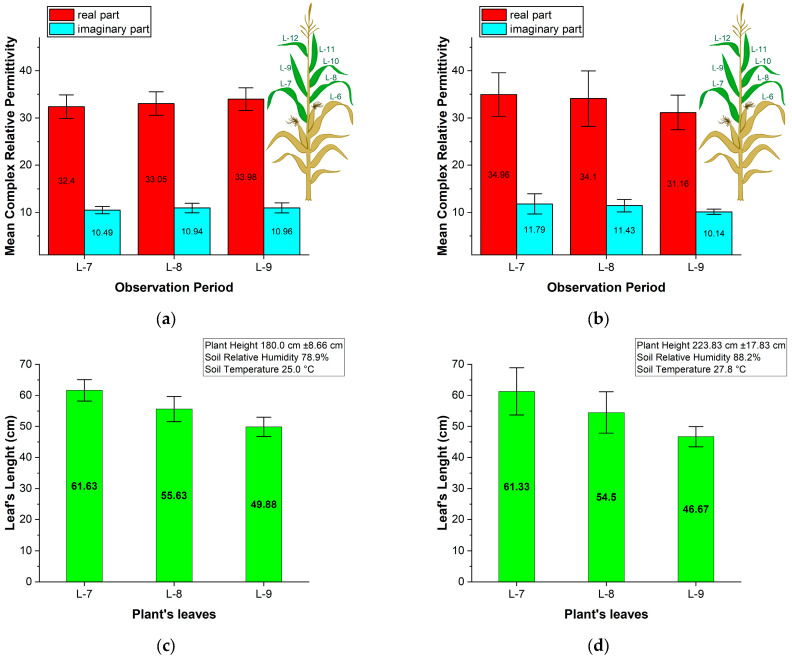
Results from measurements of corn leaves during the R5 growth stage: (**a**) Mean value and standard deviation of real and imaginary parts of corn leaf relative permittivity measured in agricultural fields near Mezdra; (**b**) Mean value and standard deviation of real and imaginary parts of corn leaf relative permittivity measured in agricultural fields near Dabrava; (**c**) Leaf length, plant height, soil relative humidity and temperature for measurements in agricultural fields near Mezdra; (**d**) Leaf length, plant height, soil relative humidity and temperature for measurements in agricultural fields near Dabrava.

Figure 7a,b show the results of measuring the real and imaginary parts of the relative permittivity in corn leaves during the R5 growth stage, conducted in two agricultural fields near Mezdra and Dabrava (Figure 2). To assess the electromagnetic properties of corn leaves under harsh environmental conditions, specifically drought, we randomly selected ten corn plants from each agricultural field, ensuring that each plant had at least three green leaves. We labelled the leaves according to their position on the plant stalk and their proximity to the soil. The first leaf closest to the soil was labelled L-1, the second leaf above it was labelled L-2, and so forth. During our measurements, the corn plants were in the R5 (Dent) growth stage, characterized by dented kernels at the top of the ear, while the first six leaves (from L-1 to L-6) had dried out. We measured the relative complex permittivity of the green leaves labelled L-7, L-8, and L-9 from ten randomly chosen corn plants in each agricultural field. We also collected data on plant height and leaf length. Additionally, we displayed the standard deviation in Figure 7 to illustrate variability and repeatability.

Figure 7a,b display the mean value and standard deviation of the real and imaginary parts of corn leaf relative permittivity measured in agricultural fields near Mezdra and Dabrava, respectively. Comparing the results from Figure 7a,b, we can conclude that the differences in the real and imaginary parts of the measured relative permittivity are negligible, regardless of the location (agricultural field) or the position of the leaves above the soil.

The *ε_r_*′ for L-7, L-8, and L-9 corn leaves is very similar across different locations. In the agricultural field near Mezdra, the mean value is 32.39 for L-7, 33.05 for L-8, and 33.98 for L-9. In the Dabrava field, the recorded mean value is 34.96 for L-7, 34.10 for L-8, and 31.16 for L-9. The standard deviation for the real part of the relative permittivity measurements ranged from 2.38 to 2.48 near Mezdra, while near Dabrava, it ranged from 3.66 to 5.86. Comparing Figure 7c,d, we see more variation in the corn plants’ height and leaf length in the agriculture field near Dabrava, which may be one of the reasons for the higher variation in the measured complex permittivity in this field.

Furthermore, the results for *ε_r_*′ are consistent with the previously obtained results [28] for corn leaves permittivity at 78% soil moisture content. We also observed that the location (agriculture field) and position of the corn leaves above the soil have little impact on the *ε_r_*″ of corn leaves. These results are consistent with results in [29]. See Table 1.

### 3.3. Electromagnetic Parameters of Sunflower

The research in this subsection focuses on the evaluation of the electromagnetic properties of sunflower leaves in the seed development stage.

**Figure 8:**
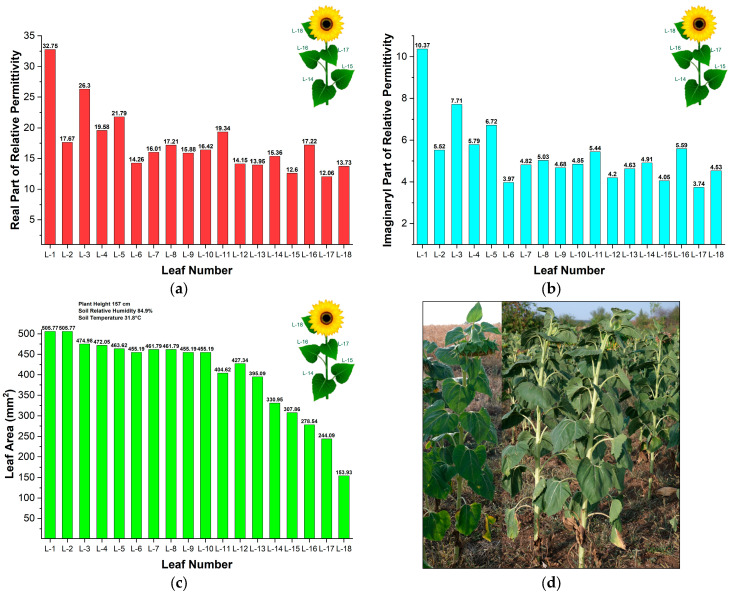
Results from measurements of sunflower leaves during the seed development stage in the agriculture field near Kameno pole: (**a**) Real part of the relative permittivity; (**b**) Imaginary part of the relative permittivity; (**c**) Leaf area, plant height, soil relative humidity and temperature; (**d**) Photos.

Figure 8a,b show the results of measured real and imaginary parts of the relative permittivity in sunflower leaves collected from the agricultural field near Kameno Pole (see Figure 2). Figure 8c,d illustrate the sunflower leaf area, environmental conditions during the measurements, and photos. In these figures, the leaves are identified based on their position on the plant stem and their distance from the soil: the first green leaf closest to the soil is labelled L-1, the second leaf above it is labelled L-2, and so forth.

We calculate the leaf area (LA) from Equation (3) according to the [30].

LA = L × W × 0.733,(3)

where L and W are the length and width of the sunflower leaf.

We observed that real and imaginary parts of relative permittivity are highest in the first leaf (L-1). Moreover, the results indicated that *ε_r_*′ varied between 37.75 and 12.06 at different leaves. Also, we observe a significant difference in *ε_r_*′ between the leaves L-1 and L-2. The epsilon is 37.75 for L-1 and 17.67 for L-2. We see the same trend for L-3 and L-4, and also for L-5 and L-6. The *ε_r_*′ is 26.30 for L-3, 21.77 for L-5, 19.58 for L-4, and 14.26 for L-6, respectively.

The results may be related to the growth pattern and sunflower leaf arrangement. As illustrated in Figure 8, sunflower leaves initially develop in opposite alternate pairs up to the middle of the stem. After this point, the leaves continue to grow as single alternate leaves along the stem until the final number of leaves is reached.

The results allow us to conclude that the leaf’s position on the plant stem and its distance from the soil significantly influence the relative permittivity’s real and imaginary parts. In the first three pairs of opposite alternate sunflower leaves (L-1 and L-2, L-3 and L-4, and L-5 and L-6), the primary leaf (the odd-numbered one) demonstrates a higher dielectric constant than the secondary leaf in each pair. Furthermore, we found that the complex permittivity of sunflower leaves L-8, L-10, L-12, L-14, L-16, and L-18 is slightly higher than that of leaves L-7, L-9, L-11, L-13, L-15, and L-17, respectively.

The results indicate that the imaginary part of the relative permittivity of a plant leaf varies with soil moisture, which in turn corresponds to the water content in the plant. This relationship can serve as an effective indicator of drought stress. Drought stress is an abiotic stressor that directly affects the health and development of plants, and its impact has been increasingly noticeable in recent years [31].

## 4. Future Work

The data obtained from this work also provide a foundation for future work on developing non-destructive sensors that can accurately monitor how plants respond to external stresses associated with climate change. For developing non-destructive sensors for plant health monitoring, we also plan to research the electromagnetic properties of plants (winter wheat, corn, etc.) in a laboratory environment under various controlled environmental conditions, for example, at different temperatures, humidity, lighting, etc. These studies will contribute to clarifying the response of plants to changing environmental conditions (external stresses). Furthermore, we plan to investigate the electromagnetic properties of wheat, corn, and other plants cultivated in various soils while maintaining constant (unchanged) environmental conditions. This research will help us understand how the agricultural environment influences the electromagnetic properties of these plants. Additionally, this knowledge will enable the development of flexible wearable antennas on biodegradable substrates sourced from plant leaves, allowing for online monitoring of plant health and reducing the e-waste generated by smart agriculture.

## 5. Conclusions

Our study results show variation in the complex permittivity of plant leaves at the frequency of 2565 MHz. We found that winter wheat and sunflower leaves have *ε_r_*′ values ranging from about 33 to 69 (winter wheat) and 13 to 32 (sunflower), respectively. In contrast, corn leaves exhibit a nearly constant *ε_r_*′ of about 33.5. We also observe that the location (agriculture field) and position of the corn leaves above the soil have little impact on the electromagnetic properties of corn leaves.

Moreover, the green leaf closest to the soil tends to have the highest value of relative complex permittivity for winter wheat and sunflower. Also, the results show that the *ε_r_*′ followed an increasing trend from the top leaf to the bottom leaf of the wheat. We also found that the *ε_r_*″ was higher in the middle layer of winter wheat leaves and is related to vertical water content distribution within winter wheat leaves.

The results allow us to conclude that the position of a leaf on the plant stem and its distance from the soil significantly influence the relative permittivity of winter wheat and sunflower. The electromagnetic properties of leaves are affected by the anatomy and morphology of plants, which play a vital role in the transport of water and nutrients. Additionally, these electromagnetic properties are influenced by the growth stages.

The data obtained on the electromagnetic parameters of different plant leaves will allow the development of non-destructive sensors that can accurately monitor how plants respond to external stresses, such as drought, associated with climate change. Furthermore, this knowledge will facilitate the online monitoring of plant health by developing flexible wearable antennas on biodegradable substrates from plant leaves without the need to extract or damage leaves.

## Figures and Tables

**Table 1:** Comparison of results from measurements of electromagnetic properties of corn leaves.

References	*ε_r_*′	*ε_r_*″	*f* ^1^
[28]	33	10	2.5
[29]	25	7	5.3
This work	33.35	10.96	2.56

^1^ The frequency (*f*) is in GHz.

## Data Availability

The data presented in this study are available on request from the authors.
